# Dendrobium officinale polysaccharide improves hair regrowth in androgenetic alopecia mice and is associated with coordinated changes in local steroid metabolism, ESR1 activation, and keratin-related follicular responses

**DOI:** 10.3389/fphar.2026.1845814

**Published:** 2026-05-18

**Authors:** Yuchen Ba, Junen Wang, Lin Qi, Zheng Liu, Dan Peng

**Affiliations:** 1 Department of Dermatology, School of the First Clinical, Hunan University of Chinese Medicine, Changsha, China; 2 Department of Dermatology, School of the Second Clinical, Hunan University of Chinese Medicine, Changsha, China; 3 Hunan University of Chinese Medicine, Changsha, China

**Keywords:** androgenetic alopecia, *Dendrobium officinale* polysaccharide, dihydrotestosterone, ESR1 activation, hair follicle, keratin-related follicular response, local steroid metabolism, proteomics

## Abstract

**Background:**

Androgenetic alopecia (AGA) is characterized by androgen-dependent follicular miniaturization and progressive disturbance of follicular structure and cycling. Local steroid metabolism, including 5α-reductase-related androgen production and aromatase-related estrogen conversion, may contribute to this process.

**Methods:**

A dihydrotestosterone (DHT)-induced mouse model of AGA was used to evaluate the *in vivo* effects of *Dendrobium officinale* polysaccharide (DOP). Dose screening was followed by histological assessment, quantitative proteomics, biochemical assays, multiplex immunofluorescence, and pharmacological perturbation with 17α-estradiol (17α-E2) and letrozole (LETZ).

**Results:**

DOP improved hair regrowth in a dose-related manner, and the high-dose group showed the most consistent changes in hair coverage, hair follicle density, dermal thickness, hair bulb diameter, and anagen proportion. Proteomic analysis served to narrow the candidate range and prioritize steroid hormone biosynthesis- and estrogen-signaling-related processes. Quantitative evaluation showed that DOP treatment was associated with lower SRD5A1 expression, higher CYP19A1 expression, lower local dihydrotestosterone and testosterone levels, increased p-ESR1, and increased KRT28/KRT71 expression. Spatial analysis further showed a shift of CYP19A1, ESR1, and KRT28 toward hair matrix- and inner root sheath-associated regions. Pharmacological modulation with 17α-E2 and LETZ altered these DOP-related changes, although not uniformly across all endpoints.

**Conclusion:**

In this model, the *in vivo* response to DOP was associated with changes related to local steroid metabolism, ESR1 activation, and keratin-related follicular responses, together with improvement in follicular morphology and cycle status. These findings support a candidate response framework for further study of DOP in AGA.

## 1 Introduction

Androgenetic alopecia (AGA) is a common, progressive, nonscarring disorder characterized by follicular miniaturization, reduced hair shaft caliber, and progressive shortening of the growth phase of the hair cycle (Oiwoh et al., 2024). Although currently available therapies can slow disease progression in some patients, their effects are often incomplete and usually require prolonged maintenance (Pozo-Pérez et al., 2024). The pathophysiology of AGA is closely linked to androgen signaling, particularly the local conversion of testosterone to dihydrotestosterone in susceptible hair follicles. However, local hormonal regulation in hair follicles is not limited to androgen excess alone. Accumulating evidence indicates that aromatase-related steroid metabolism and estrogen receptor signaling also participate in follicular homeostasis and hair-cycle regulation (Oh and Smart, 1996; Ohnemus et al., 2006), supporting the view that local steroid metabolism and receptor activity should be considered within an interconnected regulatory framework rather than as isolated processes (Yip et al., 2009).

*Dendrobium officinale* Kimura et Migo (Orchidaceae) is a traditional medicinal species, and its polysaccharides are recognized as major bioactive constituents (Chen et al., 2021; Lai et al., 2024). Previous studies have shown that Dendrobium officinale polysaccharide (DOP) can improve androgen-related hair loss in mice and promote angiogenesis, follicle regeneration, and hair growth through WNT-related mechanisms (Zhang et al., 2023; Zhang et al., 2024). Nevertheless, whether these effects are accompanied by coordinated alterations in local steroid metabolism, ESR1-related signaling, and downstream follicular structural responses remains insufficiently defined. In this context, inner root sheath-associated keratins such as KRT28 and KRT71 may provide structurally relevant downstream readouts because they are closely related to follicular differentiation and organization (Langbein et al., 2006; Jacinto et al., 2021). Accordingly, the present study was designed to investigate the effects of DOP in a DHT-induced mouse model of AGA, with particular attention to its association with local steroid metabolism, ESR1 activation, and keratin-related follicular responses.

## 2 Materials and methods

### 2.1 Experimental animals

Male C57BL/6 mice (6 weeks old, SPF grade, 16–18 g) were obtained from Hubei Beiente Biotechnology Co., Ltd. (Animal Certificate No. 4225MA48QW3P408681; Li-cense No. SCXK(E)2021-0027). Mice were housed under standard conditions (26 °C ± 1 °C, 40%–70% humidity, 12 h light/dark cycle) with free access to food and water. All animal procedures were approved by the Laboratory Animal Ethics Committee of Hunan University of Chinese Medicine (Approval No. HNUCM21-2508-03; approval date: 8 June 2025).

### 2.2 Induction of the androgenetic alopecia (AGA) model

After dorsal depilation over an area of 2 × 3 cm, mice in all groups except the control group were subjected to model induction. Intraperitoneal modeling began on the third day after depilation, after confirmation of intact pink skin and synchronized telogen status. The androgenetic alopecia (AGA) model was established using a DHT-based intraperitoneal dosing regimen under the present experimental conditions (Fu et al., 2021; Anjum et al., 2024). These studies are cited here as examples of androgen-based murine alopecia modeling strategies rather than as exact procedural equivalents to the present protocol. This regimen was selected to produce stable androgen-dependent hair-growth inhibition in this experimental setting. Dihydrotestosterone (DHT; 0.1 mg/mL, M6033, AbMole, United States) was administered by daily intraperitoneal injection at 0.2 mL per mouse for 21 consecutive days. A DHT stock solution was prepared by dissolving lyophilized DHT powder in DMSO at 10 mg/mL. The working solution (0.1 mg/mL) was freshly prepared before use by mixing 50 μL stock solution, 450 μL DMSO, 2 mL PEG300, 250 μL Tween 80, and 2.25 mL NaCl, following the reagent supplier’s recommended preparation scheme. All liquid components were passed through a 0.22 μm sterile filter during preparation. Mice received 200 μL of the working solution by daily intraperitoneal injection. During injection, mice were anesthetized, and a 34G needle was used to reduce injection-related discomfort. A dedicated vehicle-injected intraperitoneal control was not included in the present study design, and no dedicated toxicity experiment was performed. Therefore, tolerability was assessed by routine observation during the modeling period rather than by a formal toxicological endpoint.

### 2.3 Experimental grouping and intervention

In the preliminary dose-screening experiment, 5% minoxidil was used as a positive control, and a commercially obtained *D. officinale* polysaccharide (DOP) product (DOP; UV ≥ 85%, D409218, Solarbio, Beijing, China) was applied topically at different concentrations. According to the manufacturer’s certificate of analysis, the preparation was derived from the stems of *D. officinale* Kimura et Migo, authenticated by the supplier before commercial release, and provided as a standardized polysaccharide preparation with a UV-determined purity of ≥85%. For topical administration, DOP was dissolved in purified water and applied once daily at 0.1 mL per mouse to the modeled dorsal area (2 × 3 cm). A dedicated topical vehicle control was not included in the present study design. Based on gross and histological evaluation, 7.5 mg/mL DOP was selected for subsequent exploratory mechanistic analyses. Skin tissues from this stage were used for proteomic analysis.

For the mechanistic study, mice were randomly assigned to seven groups (n = 10 per group): Control, Model, DOP, 17α-E2, LETZ, DOP+ 17α-E2, and DOP + LETZ. Mice in the 17α-E2 and DOP+ 17α-E2 groups received feed containing 17α-estradiol (14.4 ppm). Mice in the LETZ and DOP + LETZ groups received letrozole (HY-14248, Med Chem Express, USA) by oral gavage at 6 mg/kg/day. Mice in the DOP-containing groups received topical DOP (7.5 mg/mL, dissolved in purified water) at 0.1 mL per mouse once daily on the modeled dorsal area. Experimental diets were prepared by Jiangsu Xietong Pharmaceutical Bio-engineering Co., Ltd. (Jiangsu, China).

### 2.4 Histological and multiplex immunofluorescence staining

At the end of treatment, dorsal skin tissues were collected, fixed in 4% paraformaldehyde for 24 h, paraffin-embedded, and sectioned at 5 μm. H&E staining was performed to evaluate hair follicle morphology and hair cycle status.

Multiplex immunofluorescence was designed to assess the spatial relationship among a steroid-metabolism-related marker (CYP19A1), a receptor-related marker (ESR1), and one representative keratin-related follicular readout (KRT28) within the same tissue context. For multiplex immunofluorescence, sections were deparaffinized, rehydrated, subjected to microwave antigen retrieval, treated with 3% hydrogen peroxide, and blocked with 10% goat serum. Sequential staining was carried out using primary antibodies against KRT28 (1:200, Cat. No. 86434-1-RR), CYP19A1 (1:500, Cat. No. 16554-1-AP), and ESR1 (1:500, Cat. No. 21244-1-AP), followed by HRP-Polymer secondary antibody (AFIHC003, AiFang Biological, Hunan, China) and tyramide signal amplification with TYR-570, TYR-520, and TYR-620 fluorophores (all 1:200). Microwave stripping and PBST washing were performed between staining cycles. Nuclei were counterstained with DAPI, and images were acquired using an eight-channel fluorescence digital slide scanner (AF-KL-20-8, AiFang Biological, China).

### 2.5 Direct data-independent acquisition proteomics analysis

Proteomic sample preparation, DIA acquisition, and primary data processing were performed through a professional DIA proteomics service workflow. For each sample, 200 ng of peptides were analyzed on a nanoElute2–timsTOF Pro2 platform (Bruker) with a nano-electrospray ion source. Peptides were separated on a PePSep C18 column (1.9 μm, 75 μm × 15 cm; Bruker, Germany) using a 45 min gradient with 0.1% formic acid in water and acetonitrile. Data were acquired in DIA-PASEF mode over an m/z range of 350–1250.

Raw MS files were processed in Spectronaut (v18.2.230802.50606) with the Pulsar search engine against the *Mus musculus* UniProt database (uniprot_Mus muscu-lus_10090_reviewed_2024.fasta). Carbamidomethylation of cysteine was set as a fixed modification, and methionine oxidation and protein N-terminal acetylation were set as variable modifications. Trypsin was used as the protease with up to two missed cleavages allowed. The false discovery rate was controlled at 1% at both the PSM and peptide levels, and precursor and fragment mass tolerances were set to 20 ppm. Protein-level differential screening and downstream pathway prioritization are described separately in the Data Processing and Statistical Analysis section.

### 2.6 Enzyme-linked immunosorbent assay (ELISA)

Skin tissue levels of dihydrotestosterone (DHT; AF030910-A), estradiol (E2; AF02566-A), and testosterone (T; AF02569-A) were measured using commercial ELISA kits (AiFang Biological, Hunan, China) according to the manufacturer’s instructions. Here, estradiol (E2) refers to the measured tissue hormone analyte rather than the 17α-E2 intervention used in the pharmacological perturbation groups. For each hormone assay, a total of 35 mouse skin tissue samples were analyzed. Absorbance was measured at 450 nm, and concentrations were calculated from the corresponding standard curves.

### 2.7 Western blot

Total protein was extracted from skin tissues and quantified by BCA assay. Equal amounts of protein were separated by SDS-PAGE and transferred to 0.45 μm nitrocellulose membranes. After blocking, membranes were incubated overnight at 4 °C with primary antibodies against SRD5A1 (Cat. No. 26001-AP, Proteintech), KRT71 (Cat. No. 26149-1-AP, Proteintech), KRT28 (Cat. No. 86434-1-RR, Proteintech), CYP19A1 (Cat. No. 16554-1-AP, Proteintech), ESR1 (Cat. No. 21244-1-AP, Proteintech), and phospho-ESR1 (Ser118; Cat. No. ET1610-32, HUABIO), each at a dilution of 1:1000. In the present study, KRT71 was assessed at the biochemical level by Western blot, whereas spatial evaluation was limited to CYP19A1, ESR1, and KRT28. GAPDH (Im-munoWay, YM3029, 1:5,000) was used as the loading control for non-nuclear proteins, whereas Histone H3 (ImmunoWay, YM3038, 1:5,000) was used as the loading control for nuclear proteins, including total ESR1 and p-ESR1. Membranes were then incubated with IR-labeled secondary antibodies, and signals were acquired using a Li-Cor Odyssey imaging system. Band intensities were quantified with ImageJ, and relative protein expression was normalized to the corresponding loading control.

### 2.8 Data Processing and Statistical Analysis

Exploratory multivariate analyses, including PCA, PLS-DA, and OPLS-DA, were performed to assess group separation and to support candidate prioritization. VIP values were derived from the OPLS-DA model and were used as an exploratory reference rather than as stand-alone inferential evidence. Based on the supplier-documented DIA proteomics analysis workflow, proteins meeting the screening criteria of *p* < 0.001 and fold change ≤0.83 or ≥1.20 were retained as candidate differentially expressed proteins (Liu et al., 2017) for downstream prioritization and biological interpretation. These candidate proteins were then subjected to volcano plot analysis, subcellular localization analysis, GO enrichment analysis, KEGG pathway analysis, and protein–protein interaction (PPI) network analysis. In the present study, the proteomics layer was used as a candidate-narrowing step to guide subsequent biochemical and tissue-level validation, rather than as a stand-alone basis for pathway-level inference.

Quantitative data are presented as mean ± SEM. Statistical analyses were performed using GraphPad Prism 10. Multiple-group comparisons were conducted by one-way ANOVA followed by Tukey’s *post hoc* test when appropriate. A two-sided *p* value <0.05 was considered statistically significant for non-proteomic quantitative assays.

## 3 Results

### 3.1 Effects of DOP on hair regrowth and hair follicle cycling in AGA mice

**FIGURE 1:**
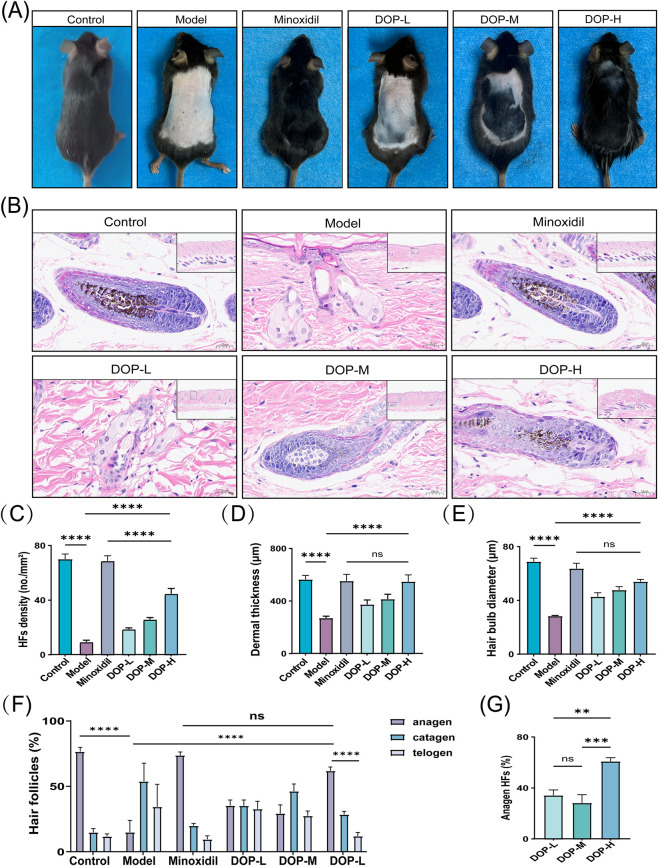
Effects of DOP on hair regrowth and hair follicle cycling in AGA mice. **(A)** Representative gross images of dorsal hair regrowth in the Control, Model, Minoxidil, and DOP-treated groups (DOP-L, 2.5 mg/mL; DOP-M, 5.0 mg/mL; DOP-H, 7.5 mg/mL). **(B)** Representative H&E-stained sections of dorsal skin from each group (n = 5). Insets show local follicular structures at higher magnification. **(C–E)** Quantitative analysis of histological parameters **(C)** hair follicle density, **(D)** dermal thickness, and **(E)** hair bulb diameter. **(F)** Relative distribution of hair follicles in the anagen, catagen, and telogen phases in each group. **(G)** Quantitative comparison of the absolute proportion of anagen-phase follicles among the DOP-L, DOP-M, and DOP-H groups. Data is presented as mean ± SEM. Statistical significance between groups is indicated as follows: *p < 0.05,**p < 0.01,***p < 0.001,****p < 0.0001; ns, not significant.

A DHT-induced mouse model of androgenetic alopecia (AGA) was used to evaluate the *in vivo* effects of DOP. As shown in Figures 1A,B, DOP improved the AGA phenotype in a dose-dependent manner. Compared with the Model group, DOP treatment increased hair follicle density, dermal thickness, and hair bulb diameter (Figures 1C–E), and increased the proportion of anagen-phase follicles (Figures 1F,G). Among the tested doses, DOP-H (7.5 mg/mL) showed the strongest overall response. The proportion of anagen-phase follicles in the DOP-H group did not differ significantly from that in the 5% Minoxidil positive-control group (Figure 1F). Based on these findings, 7.5 mg/mL was selected for subsequent exploratory mechanistic analyses.

### 3.2 Proteomics reveals global expression profile remodeling induced by DOP

**FIGURE 2:**
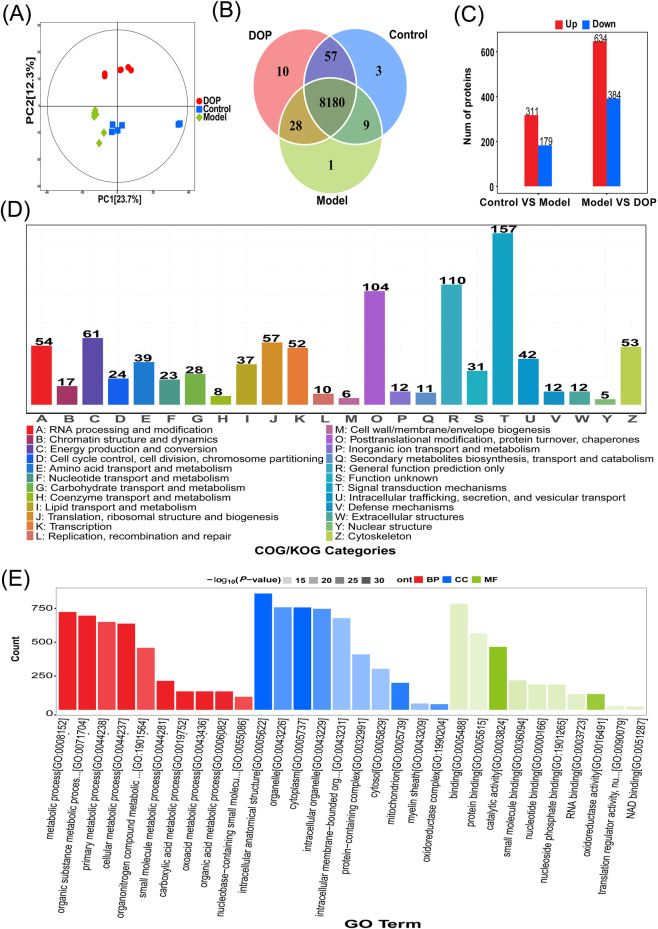
Proteomics reveals global expression profile remodeling induced by DOP. **(A)** Principal component analysis (PCA) of skin tissue samples from the Control, Model, and DOP groups (n = 6). **(B)** Venn diagram of proteins identified in the three groups. The central number indicates the proteins shared by all groups. **(C)** Numbers of candidate differentially expressed proteins in the Control vs. Model and Model vs. DOP comparisons. Red and blue bars indicate upregulated and downregulated proteins, respectively. **(D)** COG classification of candidate differentially expressed proteins in the Model vs. DOP comparison. **(E)** GO annotation of candidate differentially expressed proteins in the Model vs. DOP comparison. Red, blue, and green indicate biological process (BP), cellular component (CC), and molecular function (MF).

Quantitative proteomic analysis was performed to assess global protein expression changes in skin tissue after DOP intervention in the Control, Model, and DOP groups. PCA showed clear separation among the three groups, indicating distinct proteomic profiles under different treatment conditions (Figure 2A). According to the supplier-documented proteomics workflow, a total of 8,288 proteins were retained after preprocessing across the three groups (Figure 2B). Compared with the Model group, DOP treatment yielded 1018 candidate differentially expressed proteins, including 634 upregulated and 384 downregulated proteins, exceeding the number observed in the Control vs. Model comparison (Figure 2C) (Supplementary Table S1). COG and GO annotation showed that this candidate differentially expressed proteins were mainly associated with signal transduction, post-translational modification, protein turnover, cellular processes, metabolic processes, and biological regulation (Figures 2D,E) (Supplementary Tables S2, S3). These findings indicate that DOP intervention was associated with broad proteomic remodeling in skin tissue and that the proteomics layer provided a basis for subsequent candidate-pathway prioritization and downstream target selection.

### 3.3 Candidate pathway prioritization based on disease-related hormonal signaling

**FIGURE 3:**
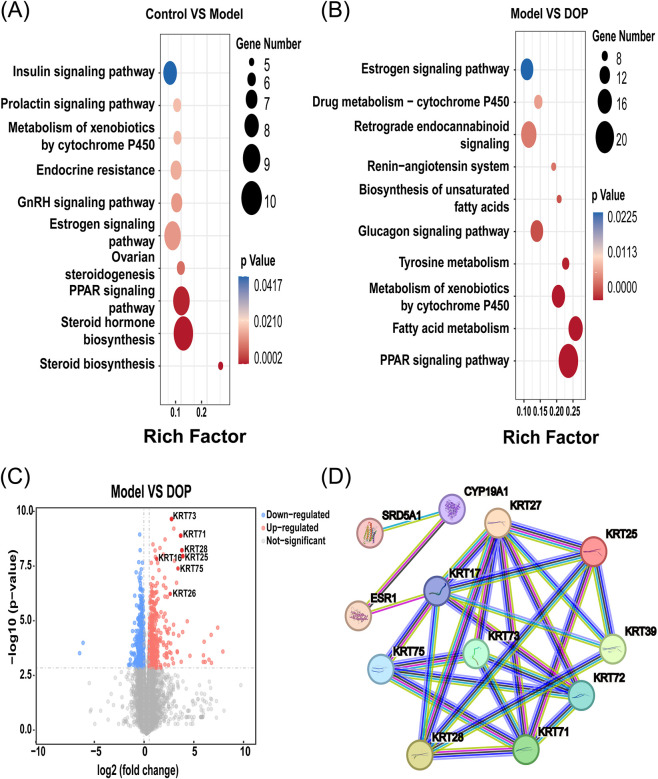
Candidate pathway prioritization based on disease-related hormonal signaling. **(A)** Partial KEGG enrichment of candidate differentially expressed proteins in the Control vs. Model comparison. **(B)** Partial KEGG enrichment of candidate differentially expressed proteins in the Model vs. DOP comparison. **(C)** Volcano plot of candidate differentially expressed proteins in the Model vs. DOP comparison. Red dots indicate upregulated candidate proteins meeting the screening criteria. KRT28, KRT71, and other selected proteins are labeled. **(D)** Protein–protein interaction (PPI) network of selected candidate proteins derived from pathway-based proteomic analysis. Nodes represent proteins, including CYP19A1, SRD5A1, ESR1, and downstream keratin family members, whereas edges represent database-supported interactions.

KEGG enrichment and PPI analyses were interpreted in the context of local steroid hormone metabolism in AGA to narrow the candidate pathways and responsive proteins. As shown in Figures 3A,B, candidate differentially expressed proteins in the Control vs. Model comparison were enriched in steroid hormone biosynthesis and estrogen signaling (Supplementary Table S4), whereas estrogen signaling remained enriched in the Model vs. DOP comparison (Supplementary Table S5). In the volcano plot, several keratin family proteins were upregulated after DOP treatment, with KRT28 and KRT71 showing the most apparent changes (Figure 3C). PPI analysis placed SRD5A1, CYP19A1, ESR1, and keratin family members within the same candidate interaction context (Figure 3D). Based on these findings, subsequent validation was prioritized toward readouts related to steroid hormone biosynthesis and downstream estrogen signaling.

### 3.4 Biochemical readouts related to steroid metabolism, ESR1, and keratin expression in the control, model, and DOP groups

**FIGURE 4:**
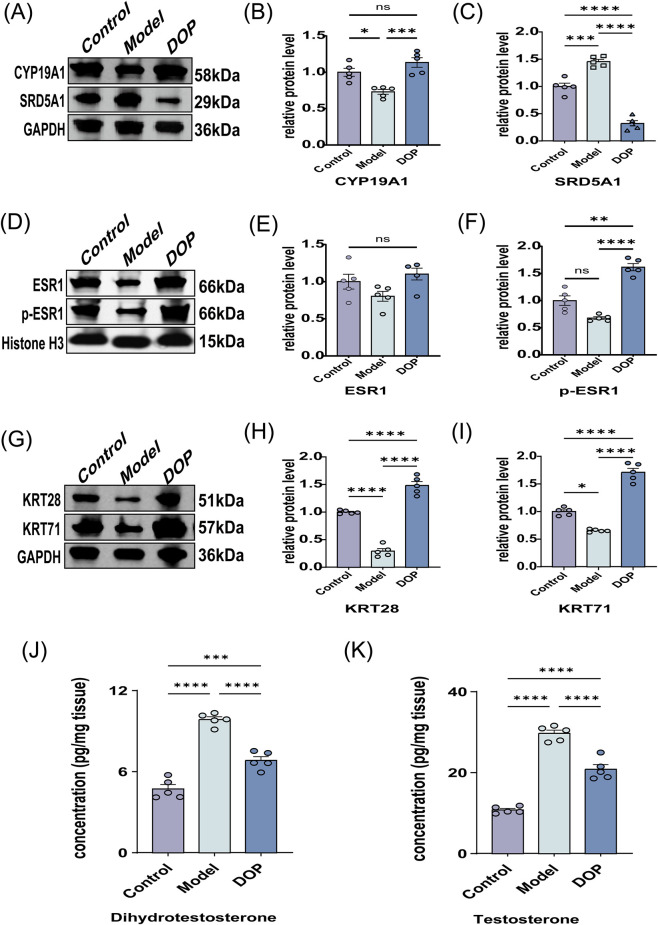
Biochemical analyses support changes related to steroid metabolism, ESR1, and keratin expression associated with the DOP response. **(A)** Representative Western blot bands showing CYP19A1 and SRD5A1 protein expression in skin tissue from each group (n = 3), with GAPDH as the loading control. **(B,C)** Quantification of relative CYP19A1 **(B)** and SRD5A1 **(C)** protein abundance. **(D)** Representative Western blot bands showing total ESR1 and phosphorylated ESR1 (p-ESR1) protein expression in skin tissue from each group (n = 3), with Histone H3 as the loading control. **(E,F)** Quantification of relative ESR1 **(E)** and p-ESR1 **(F)** protein abundance. **(G)** Representative Western blot bands showing KRT28 and KRT71 protein expression in skin tissue from each group (n = 3), with GAPDH as the loading control. **(H,I)** Quantification of relative KRT28 **(H)** and KRT71 **(I)** protein abundance. **(J,K)** ELISA-based quantification of dihydrotestosterone (DHT) **(J)** and testosterone (T) **(K)** levels in skin tissue. Data is presented as mean ± SEM. Statistical significance between groups is indicated as follows: *p < 0.05, **p < 0.01, ***p < 0.001, ****p < 0.0001; ns, not significant.

Quantitative analyses were then performed for steroid-metabolizing enzymes, local hormone levels, receptor activation status, and downstream structural proteins in skin tissue. Compared with the Control group, the Model group showed higher SRD5A1 expression, lower CYP19A1 expression, and increased DHT and T levels. After DOP intervention, these changes were partially reversed, with reduced SRD5A1 expression, increased CYP19A1 expression, and lower DHT and T levels (Figures 4A–C,J,K). Total ESR1 protein abundance did not differ significantly among groups, whereas phosphorylated ESR1 (p-ESR1) was increased in the DOP group relative to the Model group (Figures 4D–F). KRT28 and KRT71 were reduced in the Model group and increased after DOP intervention (Figures 4G–I). Overall, these findings are consistent with the involvement of changes related to steroid metabolism, ESR1, and keratin expression in the DOP-associated response, but do not by themselves establish a fully resolved linear pathway.

### 3.5 *In situ* localization and quantitative immunofluorescence analysis of CYP19A1, ESR1, and KRT28

**FIGURE 5:**
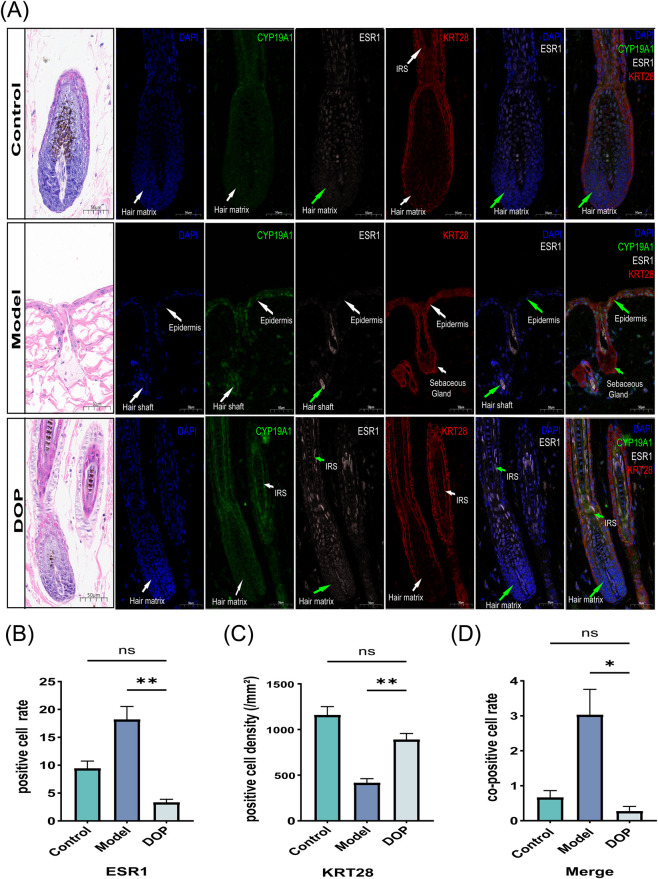
*In situ* localization and quantitative immunofluorescence analysis of CYP19A1, ESR1, and KRT28. **(A)** Representative multiplex immunofluorescence images of skin tissue from each group (n = 3). Individual fluorescence channels show the distribution of CYP19A1, ESR1, and KRT28, and DAPI was used to label nuclei. In the Control and DOP groups, the indicated signals were mainly localized to the hair matrix and inner root sheath (IRS), whereas in the Model group, signal distribution was more frequently observed in the epidermis, hair shaft, and sebaceous gland regions. **(B–D)** Quantitative analysis based on immunofluorescence images **(B)** ESR1-positive cell rate, **(C)** density of KRT28-positive cells, and **(D)** co-positive cell rate in the Merge channel. Data is presented as mean ± SEM. Statistical significance between groups is indicated as follows: *p < 0.05,**p < 0.01,***p < 0.001,****p < 0.0001; ns, not significant.

Multiplex immunofluorescence staining was performed in the Control, Model, and DOP groups to examine the spatial distribution of CYP19A1, ESR1, and KRT28 in skin tissue. Representative images showed distinct localization patterns of CYP19A1, ESR1, and KRT28 among groups. In the Control and DOP groups, the signals were mainly localized to the hair matrix and inner root sheath, whereas in the Model group they were more frequently observed in the epidermis, hair shaft, and sebaceous gland regions (Figure 5A). Quantitative analysis showed that the ESR1-positive cell rate was higher in the Model group than in the DOP group, with no significant difference between the Control and DOP groups (Figure 5B). The density of KRT28 positive cells was higher in the DOP group than in the Model group, with no significant difference between the Control and DOP groups (Figure 5C). The co-positive cell rate in the Merge channel showed a similar pattern, with a higher value in the Model group than in the DOP group and no significant difference between the Control and DOP groups (Figure 5D). These findings indicate that DOP treatment was associated with changes in the spatial distribution of CYP19A1, ESR1, and KRT28, together with selected tissue-level quantitative indices.

### 3.6 Quantitative assessment of follicular morphology and cycling under pharmacological perturbation with 17α-E2 and letrozole

**FIGURE 6:**
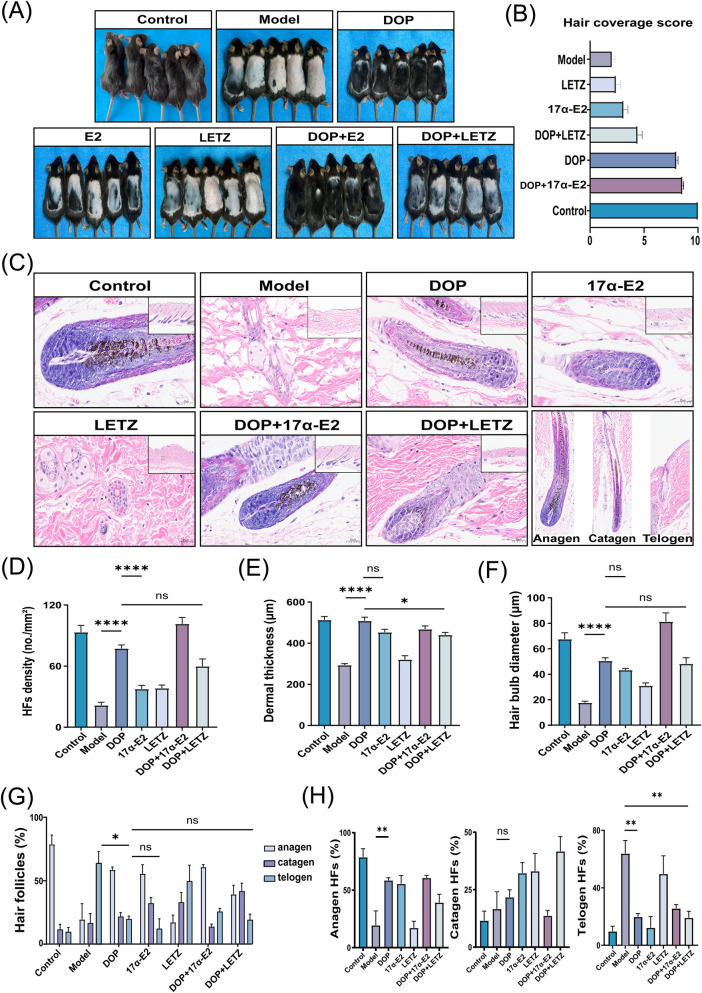
Quantitative assessment of hair follicle morphology and cycle distribution under pharmacological perturbation with 17α-E2 and letrozole. **(A)** Representative gross images of dorsal hair coverage in each group. **(B)** Hair coverage scores for each group. **(C)** Representative H&E-stained sections from each group, together with reference images for the morphological classification of anagen, catagen, and telogen follicles. **(D–F)** Quantitative analysis of follicular morphological parameters **(D)** hair follicle density, **(E)** dermal thickness, and **(F)** hair bulb diameter. **(G,H)** Quantitative analysis of follicle cycle distribution **(G)** overall distribution of follicles in the anagen, catagen, and telogen phases in each group; and **(H)** phase-specific comparison of anagen, catagen, and telogen proportions. Data is presented as mean ± SEM. Statistical significance between groups is indicated as follows: *p < 0.05,**p < 0.01,***p < 0.001,****p < 0.0001; ns, not significant.

Pharmacological perturbations with 17α-E2 and letrozole (LETZ) were introduced to assess whether the DOP-associated follicular phenotype was sensitive to endocrine perturbation within the candidate framework. Gross observation and hair coverage scoring showed more evident dorsal hair regrowth in the DOP and DOP+17α-E2 groups, whereas the DOP + LETZ group showed a lower extent of hair coverage than the DOP group (Figures 6A,B). H&E staining also showed differences in follicular morphology and cycle distribution among groups (Figure 6C).

Quantitative analysis showed that hair follicle density, dermal thickness, and hair bulb diameter were higher in the DOP group than in the Model group. Hair follicle density was also higher in the DOP group than in the 17α-E2 group, whereas dermal thickness and hair bulb diameter did not differ significantly between the two groups. Compared with DOP alone, co-treatment with LETZ was associated with reduced dermal thickness, while hair follicle density and hair bulb diameter did not differ significantly (Figures 6D–F).

Analysis of follicle cycle distribution showed that the telogen proportion was higher in the Model group than in the DOP group. In phase-specific comparisons, the Model group showed a lower anagen proportion than the DOP group and a higher telogen proportion than both the DOP and DOP + LETZ groups, whereas the catagen proportion did not differ significantly among groups (Figures 6G,H). These findings indicate that the DOP-associated follicular phenotype was sensitive to endocrine perturbation, although the resulting changes in morphology and cycling were partial and non-uniform across endpoints.

### 3.7 Quantitative evaluation of readouts related to steroid metabolism, ESR1, and keratin expression under pharmacological perturbation

**FIGURE 7:**
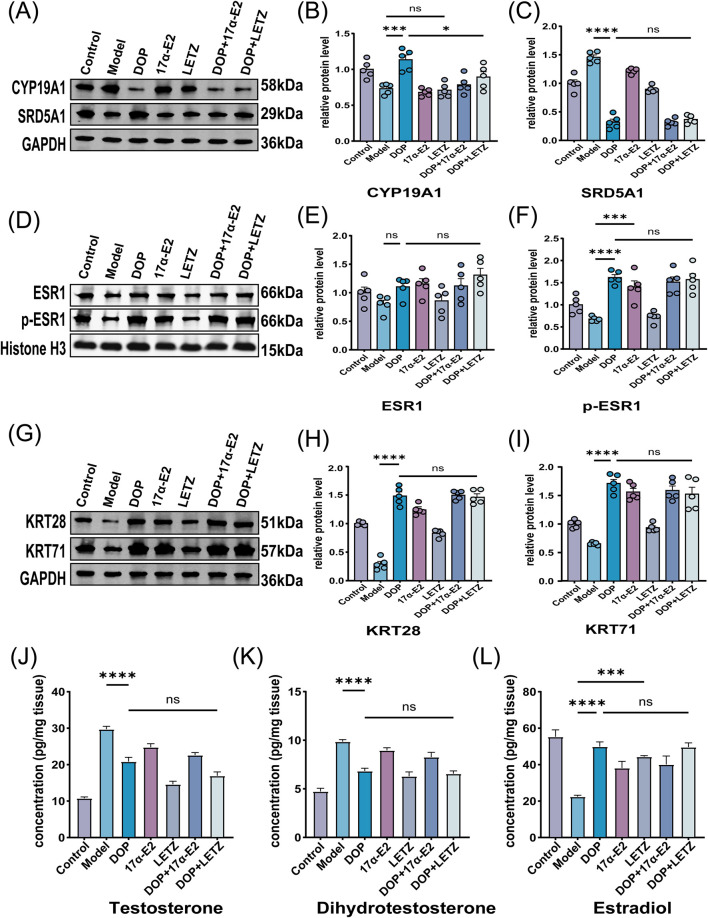
Quantitative evaluation of readouts related to steroid metabolism, ESR1, and keratin expression under pharmacological perturbation. **(A)** Representative Western blot bands of CYP19A1 and SRD5A1 in skin tissue from each group (n = 3), with GAPDH as the loading control. **(B,C)** Quantification of relative CYP19A1 **(B)** and SRD5A1 **(C)** protein levels. **(D)** Representative Western blot bands of total ESR1 and phosphorylated ESR1 (p-ESR1), with Histone H3 as the loading control (n = 3). **(E,F)** Quantification of relative ESR1 **(E)** and p-ESR1 **(F)** protein levels. **(G)** Representative Western blot bands of KRT28 and KRT71, with GAPDH as the loading control (n = 3). **(H,I)** Quantification of relative KRT28 **(H)** and KRT71 **(I)** protein levels (J–L) ELISA-based quantification of local hormone levels in skin tissue: testosterone (T) **(J)**, dihydrotestosterone (DHT) **(K)**, and tissue estradiol (E2) **(L)**. Data is presented as mean ± SEM. Statistical significance between groups is indicated as follows: *p < 0.05,**p < 0.01,***p < 0.001,****p < 0.0001; ns, not significant.

Readouts related to steroid metabolism and local hormone levels were quantified across the intervention groups. Compared with the Model group, the DOP group showed higher CYP19A1 expression and lower SRD5A1 expression. Total ESR1 did not differ significantly among groups, whereas p-ESR1, KRT28, and KRT71 were higher in the DOP group than in the Model group (Figures 7A–I). Under co-treatment with LETZ, CYP19A1 was lower than in the DOP group, while the remaining protein readouts did not differ significantly between the DOP and DOP + LETZ groups (Figures 7B–I).

Local hormone measurements showed that T and DHT levels were higher in the Model group than in the DOP group. By contrast, tissue estradiol (E2) levels were lower in the Model group than in both the DOP and LETZ groups. No significant differences were detected between the DOP and DOP + LETZ groups in T, DHT, or tissue E2 levels (Figures 7J–L). These results indicate that the DOP-associated quantitative response was sensitive to endocrine perturbation, although the changes across readouts related to steroid metabolism, ESR1, and keratin expression were partial and non-uniform.

### 3.8 *In situ* spatial evaluation of CYP19A1, ESR1, and KRT28 under pharmacological perturbation

**FIGURE 8:**
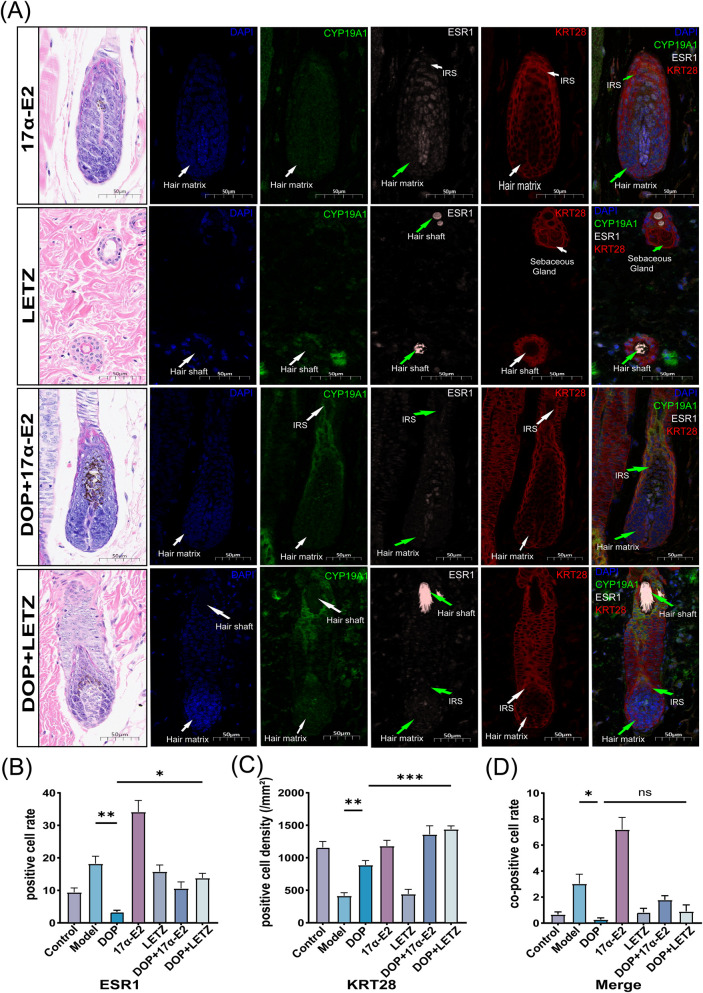
*In situ* spatial evaluation of CYP19A1, ESR1, and KRT28 under pharmacological perturbation. **(A)** Representative multiplex immunofluorescence images of skin tissue from the 17α-E2, LETZ, DOP+ 17α-E2, and DOP + LETZ groups. In the 17α-E2 and DOP+ 17α-E2 groups, the indicated signals were mainly localized to the hair matrix and inner root sheath. In the LETZ group, signals were more frequently observed in the hair shaft and sebaceous gland regions, whereas in the DOP + LETZ group, signals showed a mixed distribution pattern involving both hair matrix/inner root sheath and hair shaft-associated regions (n = 3) Scale bar = 50 μm. **(B–D)** Quantitative analysis based on multiplex immunofluorescence images: ESR1-positive cell rate **(B)**, density of KRT28-positive cells **(C)**, and co-positive cell rate in the Merge channel **(D)**. Data is presented as mean ± SEM. Statistical significance between groups is indicated as follows: *p < 0.05,**p < 0.01,***p < 0.001,****p < 0.0001; ns, not significant.

Multiplex immunofluorescence was used to examine whether the DOP-associated spatial distribution pattern of CYP19A1, ESR1, and KRT28 was sensitive to endocrine perturbation. In the 17α-E2 and DOP+17α-E2 groups, CYP19A1, ESR1, and KRT28 signals were mainly localized to the hair matrix and inner root sheath. In the LETZ group, the distribution was more superficial and more often involved the hair shaft and sebaceous gland regions. The DOP + LETZ group showed an intermediate pattern, with signals present in both deep follicular regions and hair shaft-associated areas (Figure 8A).

Quantitative analysis showed that the ESR1-positive cell rate was higher in the Model group than in the DOP group and was also higher in the DOP + LETZ group than in the DOP group (Figure 8B). The density of KRT28-positive cells was higher in the DOP group than in the Model group and was also higher in the DOP + LETZ group than in the DOP group (Figure 8C). In the Merge channel, the co-positive cell rate was higher in the Model group than in the DOP group, whereas no significant difference was detected between the DOP and DOP + LETZ groups (Figure 8D). These results indicate that the DOP-associated spatial response was sensitive to endocrine perturbation, although the resulting changes in tissue distribution and selected quantitative indices of CYP19A1, ESR1, and KRT28 were non-uniform.

## 4 Discussion

In this study, DOP was evaluated in a DHT-induced mouse model of AGA at the phenotypic, histological, proteomic, biochemical, spatial, and pharmacological levels. DOP improved hair regrowth in a dose-related manner, and the high-dose group showed the most consistent changes in hair coverage, follicle density, dermal thickness, hair bulb diameter, and anagen proportion. These findings extend earlier reports that DOP promotes hair regrowth and follicular regeneration in androgen-related experimental settings (Zhang et al., 2023; Zhang et al., 2024). In the present work, the response to DOP was not confined to gross hair recovery but was accompanied by changes in follicular structure and cycle status. Accordingly, the present study was designed not only to document the phenotypic effect of DOP, but also to examine whether the DOP-associated response was linked to local steroid metabolism, ESR1-related signaling, and downstream keratin-related follicular changes. At the same time, the present data are interpreted at the level of a candidate response framework rather than a fully resolved linear mechanism.

The proteomic data indicates that DOP treatment was associated with broader remodeling of the skin protein profile rather than isolated shifts in a small number of terminal readouts. Given the hormone-dependent pathological background of AGA (Chen S. et al., 2025; Liu et al., 2025; Ohnemus et al., 2006; Yip et al., 2009; Owecka et al., 2024; Song et al., 2025) the enriched pathways were not interpreted with equal weight. Instead, the analysis was narrowed to steroid hormone biosynthesis and downstream estrogen signaling, because these pathways were more directly connected to the disease context and to the biology of the hair follicle microenvironment (Oh and Smart, 1996; Conrad and Paus, 2004; Ohnemus et al., 2006; Yip et al., 2009; Sánchez et al., 2018; Sánchez et al., 2023; Charoensuksira et al., 2024; Li et al., 2024a; Owecka et al., 2024; Dorzweiler, 2025; Nai et al., 2025). In this part of the study, proteomics served primarily to reduce the candidate range for interpretation of the DOP-associated. Accordingly, the proteomics layer was used as a candidate-narrowing and target-prioritization step, rather than as a stand-alone basis for pathway-level inference. This use of global molecular profiling consists of broader multi-omics and proteomic work, where such datasets are informative for candidate selection but do not by themselves establish causal hierarchy (Langbein et al., 2006; Fujimoto et al., 2012; Jacinto et al., 2021; Charoensuksira et al., 2024; Li et al., 2024a; Nai et al., 2025).

The quantitative data further refined interpretation of the DOP-associated response. Relative to the Model group, the DOP group showed higher CYP19A1 expression and lower SRD5A1 expression, together with lower local testosterone and dihydrotestosterone levels. Total ESR1 did not change significantly, whereas p-ESR1 increased after DOP treatment. This pattern places the DOP response closer to adjustment of local steroid metabolism and receptor activation status than to simple changes in total receptor abundance (Ohnemus et al., 2006; Yip et al., 2009; Sánchez et al., 2018; Sánchez et al., 2023; Ober-Reynolds et al., 2023; Chen Q. et al., 2025; Lee et al., 2025). It also accords with the view that aromatase-related metabolism and downstream estrogen-responsive signaling may participate in follicular homeostasis alongside androgen-dependent processes (Oh and Smart, 1996; Conrad and Paus, 2004; Duan et al., 2021; Ober-Reynolds et al., 2023; Chen Q. et al., 2025; Lee et al., 2025). Under co-treatment with LETZ, CYP19A1 decreased relative to DOP alone, whereas the remaining protein and hormone readouts did not shift uniformly. These data indicate that the DOP-associated quantitative response was sensitive to perturbation of local estrogen-related metabolic signaling, but the resulting pattern was partial and non-uniform rather than consistent with a uniform reversal model.

The spatial data provided an additional level of information for interpretation of the DOP-associated response. KRT28 and KRT71 are closely related to inner root sheath structure and follicular organization (Bawden et al., 2001; Tanaka et al., 2007; Kuramoto et al., 2010; Kauffman et al., 2015; Li et al., 2024b; Li et al., 2026). In the present study, however, spatial evaluation was limited to CYP19A1, ESR1, and KRT28, whereas KRT71 was assessed at the biochemical level only. CYP19A1, ESR1, and KRT28 signals in the Control and DOP groups were mainly localized to the hair matrix and inner root sheath, whereas the Model group showed a more superficial distribution involving the epidermis, hair shaft, and sebaceous gland regions. After endocrine perturbation, the 17α-E2 and DOP+17α-E2 groups retained a deeper follicular distribution pattern, whereas the LETZ group showed a more superficial pattern and the DOP + LETZ group showed an intermediate distribution. These findings suggest that the AGA state involved not only quantitative shifts in candidate proteins, but also altered localization patterns within the follicular microenvironment (Langbein et al., 2006; Tanaka et al., 2007; Jacinto et al., 2021; Song et al., 2025). The increase in KRT28 after DOP treatment, together with the shift toward hair matrix- and inner root sheath-associated regions, supports a link between upstream steroid-related changes and downstream structural follicular responses, whereas KRT71 provided biochemical support at the protein-expression level.

Overall, the results support a candidate response framework in which the *in vivo* response to DOP was associated with changes in local steroid metabolism, ESR1 activation, and keratin-related follicular responses. More specifically, DOP treatment was accompanied by lower SRD5A1, higher CYP19A1, reduced local DHT and testosterone, increased p-ESR1, increased KRT28 and KRT71, and shift toward hair matrix- and inner root sheath-associated localization for CYP19A1, ESR1, and KRT28. These changes were accompanied by improvement in follicular morphology and cycle status. However, the present data do not justify describing this framework as a fully established single linear pathway. A more appropriate interpretation is that these processes participate together in the DOP response, whereas the internal hierarchy and causal order remain unresolved.

The pharmacological perturbation experiments further refined interpretation of the DOP-associated response. Rather than serving as stand-alone evaluations of 17α-E2 or LETZ, these perturbation analyses were used to test whether the DOP-responsive phenotype was sensitive to changes in local estrogen-related metabolic and signaling processes. The effect was detectable, but it was not uniform across all endpoints. This is consistent with the view that CYP19A1-related metabolic regulation and downstream estrogen signaling are involved in the DOP response (Ohnemus et al., 2006; Owecka et al., 2024; Chen Q. et al., 2025; Lee et al., 2025), while also indicating that the present data do not support reduction of the response to a single molecular node. The separation of Figures 7, 8 is therefore analytically useful: some changes were more evident at the level of total abundance, whereas others were clearer at the level of tissue distribution.

Several limitations should be noted. The study relied mainly on endpoint tissue analyses and therefore does not establish temporal causality. In addition, because dedicated vehicle-injected intraperitoneal control and a dedicated topical vehicle control were not included, potential injection-related stress and vehicle-related effects cannot be fully excluded. The proteomics layer was used primarily for candidate narrowing and target prioritization, while pharmacological perturbation provided supportive evidence for functional relevance; neither approach replaces direct validation through targeted perturbation of key nodes. Both 17α-E2 and LETZ were systemic interventions, so the present data are better interpreted as evidence of functional relevance than as proof of pathway identity (Yokota et al., 2025). Spatial evaluation was limited to CYP19A1, ESR1, and KRT28, whereas KRT71 was assessed at the biochemical level only. Moreover, because minoxidil was used only in the preliminary dose-screening stage and was not carried through the downstream molecular analyses, the present study cannot determine whether the observed molecular pattern is specific to DOP or reflects a broader hair-regrowth rescue-associated response. Finally, the study was conducted mainly at the tissue level and did not resolve the contribution of specific cell populations. Further work should combine temporal analysis with cell-specific and spatially resolved approaches to better define the relationship among CYP19A1, ESR1 activation, and keratin-related follicular responses (Langbein et al., 2006; Fujimoto et al., 2012; Li et al., 2024a).

## 5 Conclusion

In a DHT-induced mouse model of AGA, DOP improved hair regrowth, with the most consistent effect observed at the high dose. Proteomic analysis served to narrow the candidate range and prioritize steroid hormone biosynthesis- and estrogen-signaling-related processes. Quantitative analyses and spatial evaluation indicated that the *in vivo* response to DOP was associated with changes related to local steroid metabolism, ESR1 activation, and keratin-related follicular responses, together with improvement in follicular morphology and cycle status. Based on the present evidence, DOP should be interpreted as being associated with this candidate framework rather than as acting through a fully established single-pathway mechanism. These findings provide a basis for further investigation of DOP in AGA.

## Data Availability

The data presented in the study are deposited in the iProX repository, ProteomeXchange accession number PXD077413.
